# Equine induced pluripotent stem cells are responsive to inflammatory cytokines before and after differentiation into musculoskeletal cell types

**DOI:** 10.1007/s11626-023-00800-3

**Published:** 2023-08-15

**Authors:** Esther Palomino Lago, Elizabeth R. Jelbert, Arabella Baird, Pak Y. Lam, Deborah J. Guest

**Affiliations:** 1 Department of Clinical Sciences and Services, The Royal Veterinary College, Hawkshead Lane, North Mymms, Hatfield, AL9 7TA Herts UK; 2 Animal Health Trust, Lanwades Park, Kentford, Newmarket, CB8 7UU UK

**Keywords:** Equine, Induced pluripotent stem cells, Inflammation, Musculoskeletal, Differentiation

## Abstract

Persistent inflammation is associated with the poor regeneration of musculoskeletal tissues. Embryonic stem cells (ESCs) have an attenuated response to inflammatory cytokines, but there are mixed reports on the response of induced pluripotent stem cells (iPSCs) to inflammation. Horses provide a relevant large animal model for studying musculoskeletal tissue diseases and the testing of novel therapies. The aim of this study was to determine if equine iPSCs are responsive to the inflammatory cytokines IL-1β, TNFα and IFN-γ in their undifferentiated state, or following differentiation into tendon and cartilage-like cells. We demonstrated that in undifferentiated iPSCs, the cytokines induce NF-κB P65 and STAT1 nuclear translocation which leads to cell death, decreased *OCT4* expression and increased expression of inflammatory genes. Following differentiation towards cartilage-like cells exposure to the cytokines resulted in STAT1 nuclear translocation, changes in cartilage gene expression and increased expression of matrix metalloproteinases (MMPs) and inflammatory genes. Exposure of iPSC-derived tendon-like cells to the cytokines resulted nuclear translocation of NF-κB P65 and STAT1, altered tendon gene expression, increased *MMP* expression and increased expression of inflammatory genes. Equine iPSCs are therefore capable of responding to inflammatory stimulation and this may have relevance for their future clinical application.

**Supplementary Information:**

The online version contains supplementary material available at 10.1007/s11626-023-00800-3.

## Introduction

Musculoskeletal injuries to tissues such as tendon and cartilage are a major cause of morbidity in humans (Badley *et al*. 1994; Zhang and Jordan 2010; Sheth *et al*. 2017). Horses also suffer from naturally occurring injuries to these tissues and provide a relevant, large animal model for their study (Ribitsch *et al*. 2020). In horses, tendon injuries and cartilage damage occur frequently (Williams *et al*. 2001; Goodrich and Nixon 2006; Avella *et al*. 2009; Russell *et al*. 2017) and are a leading cause of retirement (Lam *et al*. 2007; Shrestha *et al*. 2021). This is due to the poor natural regeneration of these tissues, formation of scar tissue and propensity to reinjure (Dyson 2004; Ribitsch *et al*. 2021). Injuries to these tissues lead to increased inflammatory cytokine production (Hosaka *et al*. 2002; KAMM *et al*. 2010; Bertuglia *et al*. 2016; Morita *et al*. 2017; Dakin *et al*. 2018b). Although some inflammation may help to initiate tissue repair, evidence suggests that the persistence of inflammation may contribute to poor tissue regeneration and subsequent fibrotic healing (Dakin *et al*. 2013, 2014, 2018a; van der Kraan 2019).

We have previously demonstrated that the inflammatory cytokine Interleukin 1 beta (IL-1β) has negative effects on tendon cell gene expression and functional ability to contract a three-dimensional (3D) collagen gel (McClellan *et al*. 2019a). In contrast, we found that horse embryonic stem cell (ESC) derived tendon cells do not respond to IL-1β (McClellan *et al*. 2019a). This is because they have very low expression of the IL-1 receptor 1 (IL1R1) and high expression of both the decoy receptor IL-1 receptor 2 (IL1R2) and IL-1 receptor antagonist protein (IL1RA) (McClellan *et al*. 2019a).

Human and mouse ESCs have also been shown to have an attenuated response to inflammatory cytokines (Allen *et al*. 2000; Guo *et al*. 2015; D’Angelo *et al*. 2017; Chen *et al*. 2020), even after differentiation to a range of cell types including osteoblasts (Sidney *et al*. 2014), fibroblasts (Chen *et al*. 2020), smooth muscle cells (Zampetaki *et al*. 2007) and endothelial cells (Zampetaki *et al*. 2007; Rajan *et al*. 2008; Glaser* et al*. 2011). The relevance of this for the clinical translation of cell products to aid tissue repair and regeneration is not clear. On the one hand, it may be of benefit if the transplanted cells do not respond to, or perpetuate local inflammation. On the other hand, mouse ESCs and ESC-derived endothelial cells fail to respond to the bacterial endotoxin lipopolysaccharide (LPS) (Zampetaki *et al*. 2006) and undifferentiated human ESCs fail to activate an anti-viral response to double stranded RNA (dsRNA) (Chen *et al*. 2010). If the cells have low innate immunity and cannot detect or respond to infection, this may be problematic following transplantation (Guo *et al*. 2015).

Induced pluripotent stem cells (iPSCs) are derived by the reprogramming of adult cells back into a pluripotent state. As the adult cells have already developed innate immunity and cytokine responsiveness, it is of interest to determine if this is “reset” during reprogramming. Mouse iPSCs have a poor antiviral response to Baculovirus infection (Chen *et al*. 2012) and introduction of double stranded RNA into the cytoplasm (Chen *et al*. 2010). While undifferentiated human iPSCs do not respond to Tumour Necrosis factor alpha (TNFα) and Interferon gamma (IFN-γ) (Chen *et al*. 2020). However, human iPSCs which have been differentiated into pancreatic beta cells (Demine *et al*. 2020), cardiomyocytes (Saraf *et al*. 2021), astrocytes (Hyvärinen *et al*. 2019), neurons (Hagman *et al*. 2019) or chondrocytes (Lee *et al*. 2017) all show some responsiveness to inflammatory cytokines including IL-1β, TNFα and IFNγ. Although in some cases, the response appears reduced compared to primary adult cells (Lee *et al*. 2017).

We have previously demonstrated that equine iPSCs can differentiate into tendon-like cells in vitro (Bavin *et al*. 2015) and the successful differentiation of equine iPSCs to chondrocytes has been reported by others (Quattrocelli *et al*. 2016). Recently, we have shown that in adult tissue derived tendon cells, a combination of inflammatory cytokines (IL-1β, TNFα and IFN-γ) has an additive effect in inducing more damaging consequences for tendon cell gene expression and function (Smith *et al*. 2023). Here we aim to determine if equine iPSCs are responsive to these inflammatory cytokines either in their undifferentiated state or following differentiation towards tendon and chondrocyte-like cells.

## Materials and Methods

**Figure 1.**
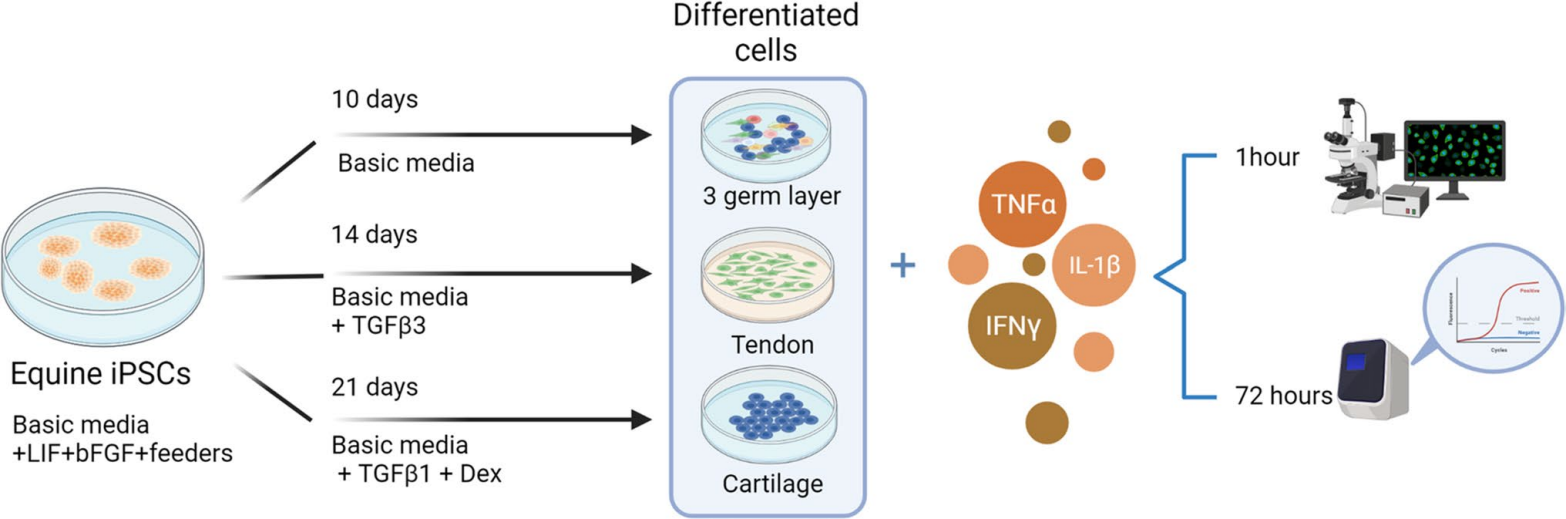
Overview of the experimental approach. (Figure created using BioRender.com).

An overview of the experimental approach used in this study is shown in Fig. 1.

### Equine iPSC culture

Three lines of induced pluripotent stem cells (iPSCs) were generated from equine skin fibroblasts by retroviral transduction using methods previously described (Bavin *et al*. 2015; Baird *et al*. 2018). Briefly, fibroblasts were isolated from skin biopsies of male Thoroughbred horses (age 2–4 yr) at post-mortem. All animals had been euthanised for reasons unrelated to this study and with the consent of the Animal Health Trust Ethical Review Committee (AHT_02_2012) and Royal Veterinary College Clinical Research Ethical Review Board (URN 2021 2035–2). Phoenix gag-pol packaging cells were transfected with 3 µg of pVPack-VSV-G (Agilent Technologies, Cheadle, UK) along with 3 µg of pMXs.hOct4 (Addgene 17217), pMXs.hSox2 (Addgene 17218), pMXs.hKlf4 (Addgene 17219), pMXs.hc-Myc (Addgene 17220), or pMX.GFP (Cell Biolabs, San Diego, CA). Transfections were carried out using lipofectamine 3000 and Opti-MEM media (both Invitrogen, Thermo Fisher, Horsham, UK) according to the manufacturer’s instructions. After 48 h culture supernatant containing the viral particles was pooled, filtered through a 0.45 µM filter (Nalgene, Thermo Fisher, UK), supplemented with 10 µg/ml polybrene (Sigma-Aldrich, Gillingham, UK) and used to infect equine skin fibroblasts which had been plated at a density of 1 × 10^4^ 24 h before infection. After three rounds of viral infection, at 48 h intervals, infected cells were plated at a density of 5 × 10^3^ cells per 10 cm plate pre-seeded with feeder cells (mitotically inactivated mouse embryonic fibroblasts). The media was replaced with basic media [DMEM/F12, 15% fetal bovine serum (FBS), 2 mM L-glutamine, 0.1 mM non-essential amino acids, 0.1 mM 2-βmercaptoethanol, 1 mM sodium pyruvate (all from Thermo Fisher, UK)] plus 1000 U/ml Leukemia inhibitory factor (LIF) (Preprotech, London, UK) and 10 ng/ml basic fibroblast growth factor (bFGF) (Peprotech).

iPSC basic media was replaced every 48 h until iPSC colonies began to appear and reached a large enough size to manually pick selected colonies. These colonies were used to establish clonal iPSC lines. iPSCs were mechanically passaged in the presence of 2 μM Thiazovivin (Miltenyi Biotec, Bisley, UK). iPSCs were used at passage 8–15 for all the conditions in this study.

### Equine iPSC differentiation

For embryoid body formation, the iPSCs were passaged onto low attachment plates (Corning Life Sciences, Corning, NY) in basic media without feeder cells, LIF or bFGF for up to 14 d. Equine iPSCs were allowed to spontaneously differentiate into derivatives of all three germ layers by culturing in the absence of feeder cells, in basic media (without LIF and bFGF) for 10 d, as previously described (Bavin *et al*. 2015). Equine iPSCs were directed to differentiate into tenocyte-like cells by culturing in the absence of feeder cells in basic media (without LIF and bFGF) supplemented with 20 ng/ml TGF-β3 (Peprotech) for 14 d as previously described (Bavin *et al*. 2015). Equine iPSCs were directed to differentiate into cartilage-like cells by culturing in the absence of feeder cells, in basic media (without LIF and bFGF) supplemented with 10 ng/ml TGF-β1 (Peprotech) and 100 nM dexamethasone (Sigma-Aldrich) for 21 d. In each case, media was replenished every 3–4 days.

### Immunofluorescence

iPSC cells (differentiated or undifferentiated) were cultured on gelatin-coated glass coverslips (Sigma-Aldrich) prior to stimulation with inflammatory cytokines, as described previously (Smith *et al*. 2023). Briefly, stimulation was performed with TNFα (10 ng/mL), IL-1β (1nM) and/or IFN-γ (100 ng/mL) (all PeproTech) for 1 h (Smith *et al*. 2023). Unstimulated cells served as controls. Following stimulation cells were fixed in 3% paraformaldehyde (PFA) for 20 min. Fixed cells were permeabilised with 0.1% Triton-X-100 (Sigma-Aldrich) at room temperature for 1 h and then blocked with 2.5% normal horse serum (NHS; Vector Laboratories, Peterborough, UK) for 20 min. Primary antibodies incubations were performed overnight at 4°C in NHS, followed by washing and secondary antibody incubation in NHS for 3 h at room temperature. Negative controls were performed using the secondary antibody only. Coverslips were mounted using Vectashield Hardset with DAPI (4′,6-diamidino-2-phenylindole, Vector Laboratories). Primary antibodies are shown in Table 1. The secondary antibodies were either goat anti-mouse IgG Alexa fluor 594 (Thermo Fisher, A-11005) 1:200, goat anti-rabbit IgG Alexa Fluor 594 1:200 (Thermo Fisher, A-11012) or goat anti-rat Texas Red 1:200 (Sigma-Aldrich, SAB3700668).

**Table 1.** List of primary antibodies used

Marker	Species	Clone	Dilution	Company	References
NF-κB (P65)	Mouse	Monoclonal (572)	1:100	Thermo Fisher (436700)	(Smith *et al*. 2023)
STAT1	Rabbit	Monoclonal (EPR4407)	1:200	Abcam (ab109320)	(Smith *et al*. 2023)
JNK1,2,3	Rabbit	Monoclonal (EPR16797211)	1:200	Abcam (ab179461)	(Smith *et al*. 2023)
P38 MAPK	Rabbit	Polyclonal	1:100	Cell Signalling Technology (CS9212)	(Gardner *et al*. 2016; Smith *et al*. 2023)
Oct4	Rabbit	Polyclonal	1:100	Abcam (ab18976)	(Baird *et al*. 2018)
SSEA1	Mouse	Monoclonal (MC-480)	1:100	Chemicon (MAB4301)	(Guest and Allen 2007; Bavin *et al*. 2015; Baird *et al*. 2018)
SSEA3	Rat	Monoclonal (MC-631)	1:100	Chemicon (MAB4303)	(Guest and Allen 2007; Bavin *et al*. 2015; Baird *et al*. 2018)
SSEA4	Mouse	Monoclonal (MC-813–70)	1:100	Millipore (MAB4304)	(Guest and Allen 2007; Bavin *et al*. 2015; Baird *et al*. 2018)
TRA-1–60	Mouse	Monoclonal	1:500	Kindly provided by Prof Peter Andrews at the University of Sheffield, UK	(Guest and Allen 2007; Bavin *et al*. 2015; Baird *et al*. 2018)
TRA-1–81	Mouse	Monoclonal	1:500	Kindly provided by Prof Peter Andrews at the University of Sheffield, UK	(Guest and Allen 2007; Bavin *et al*. 2015; Baird *et al*. 2018)
βIII tubulin	Mouse	Monoclonal (SDL.3D10)	1:100	Sigma-Aldrich (T8660)	(Guest and Allen 2007; Bavin *et al*. 2015; Baird *et al*. 2018)
AFP	Rabbit	Polyclonal	1:500	Biorbyt (orb7822)	(Bavin *et al*. 2015; Baird *et al*. 2018)
Muscle actin	Mouse	Monoclonal (HHF35)	1:200	Dako (M0635)	(Bavin *et al*. 2015; Baird *et al*. 2018)

Nuclear fluorescent intensity (NFI) was quantified by measuring the mean grey scale of the nucleus using ImageJ software. NFI data is presented as fold change in intensity of the inflammatory cytokine treated cells, compared with untreated controls.

### RNA extraction, cDNA synthesis and quantitative PCR

iPSCs were cultured under undifferentiated or differentiated conditions prior to stimulation with inflammatory cytokines. Stimulation was performed with TNFα (10 ng/mL), IL-1β (1 nM) and IFN-γ (100 ng/mL) for 72 h (Smith *et al*. 2023). Unstimulated cells served as controls. Following stimulation, RNA was collected using Tri-reagent (Sigma-Aldrich) and extracted using the RNeasy mini kit (Qiagen, UK) following the manufacturer’s instructions. Purified RNA was treated with the DNA-free™ DNA removal kit (Invitrogen, Thermo Fisher) according to the manufacturer’s instructions to remove genomic DNA contamination. cDNA was synthesised from 1 µg of treated RNA using a sensiFAST cDNA synthesis kit (Bioline, UK). 2 µl of cDNA (corresponding to 20 ng) was used for each quantitative PCR (qPCR) reaction. Primer sequences can be found in Table 2. qPCR was performed in duplicate using SYBR Green containing supermix (Bioline) on a Bio-rad C1000 Touch Thermal Cycler (Bio-rad, UK). PCR cycle parameters were as follows: 95°C (10 min), followed by 45 cycles of 95°C (15 s), 60°C (15 s) and 72°C (15 s). Following this, a melt curve was produced with readings taken every 1°C from 65°C to 95°C. Relative gene expression levels were normalised with the housekeeping gene 18s rRNA using the 2^−ΔΔCt^ method (Livak and Schmittgen 2001). qPCR data is presented as a fold change in gene expression of the inflammatory cytokine treated cells, compared with untreated controls.

**Table 2.** Primers used in qPCR

Gene	Protein Name	Forward Primer	Reverse Primer
*18S rRNA*	18s ribosomal RNA	CCCAGTGAGAATGCCCTCTA	TGGCTGAGCAAGGTGTTATG
*ACAN*	Aggrecan	GCGGTACGAGATCAACTCCC	GCGACAAGAAGAGGACACCA
*CNMD*	Chondromodulin	GGGAACAACTTGGAGACCTT	TTCTCTCCTCCAGCAAAACG
*COL1A1*	Collagen alpha-1(I) chain	TGCGAAGACACCAAGAACTG	GACTCCTGTGGTTTGGTCGT
*COL2A1*	Collagen alpha-2(I) chain	TCCTGGTGTCAAAGGTCACA	TCCCTTAGCACCATCCAGAC
*COMP*	Cartilage oligomeric matrix protein	AGAACATCATCTGGGCCAAC	CGCTGGATCTCGTAGTCCTC
*COX2*	Cyclooxygenase-2	CAATCGAGTGGTTCTCCCCA	GGCCACGAGAGTTGTCTGAT
*IL-1β*	Interleukin 1 beta	CTCCTCGAAGGCGCAGTC	CCACAAGACAGGTACAGGTTC
*IL6*	Interleukin 6	GAAAGAGAGCTTCATCTGCCC	ACTGGAGTGACGGTGCTTG
*MMP1*	Matrix Metallopeptidase 1 (Interstitial collagenase)	CTTTGATGGACCTGGAGGAA	GAATGGCCAAATTCATGAGC
*MMP13*	Matrix Metallopeptidase 13 (Collagenase 3)	GCCACTTTGTGCTTCCTGAT	CGCATTTGTCTGGTGTTTTG
*MMP2*	Matrix Metallopeptidase 2 (72 kDa type IV collagenase)	CAGGAGGAGAAGGCTGTGTT	AGGGTGCTGGCTGAGTAGAC
*MMP3*	Matrix Metallopeptidase 3 (Stromelysin-1)	TGGACCTGGAAAAGTTTTGG	GACCAAGTTCATGAGCAGCA
*MMP8*	Matrix Metallopeptidase 8 (Neutrophil collagenase)	TTTGATGGACCCAATGGAAT	TTCATGGGCAGCAACAATAA
*MMP9*	Matrix Metallopeptidase 9	GAGATCGGGAATCATCTCCA	CCAAGAGTCGCCAGTACCTC
*OCT4*	Octamer-binding transcription factor 4	GATATACCCAGGCCGATGTG	GTCGTTTGGCTGAACACCTT
*SCX*	Basic helix-loop-helix transcription factor scleraxis	CCCAAACAGATCTGCACCTT	ATCCGCCTCTAACTCCGAAT
*SOX9*	Transcription factor SOX-9	GCTCTGGAGACTTCTGAACGA	GTAATCCGGGTGGTCCTTCT
*STAT3*	Signal transducer and activator of transcription 3	AGTGACCAGGCAGAAGATGC	CTGTTGTCGCCTCTTCCAGT
*TGF-β1*	Transforming Growth Factor-β1	CGCGTGCTAATGGTGGAAAA	GAGCTCCGACGTGTTGAAGA
*THBS4*	Thrombospondin-4	GGGAAATGGGGTTACCTGTT	CGGGTAGCAGGGATGATATT
*TNC*	Tenascin	AACCCGTCCAAAGAGACCTT	GCGTGGGATGGAAGTATCAT
*TNFα*	Tumor necrosis factor alpha	AAAGGACATCATGAGCACTGAAAG	GGGCCCCCTGCCTTCT
*TNMD*	Tenomodulin	GTCCCTCAAGTGAAGGTGGA	CCTCGACGGCAGTAAATACAA

### Alcian blue staining and leaching

Undifferentiated equine iPSCs and iPSC-derived cartilage-like cells were fixed in 3% PFA for 20 min prior to washing and staining in Alcian blue stain solution (1% w/v in 3% acetic acid solution, pH 2.5, Sigma, B8438) following the manufacturer’s instructions. Briefly, cells were stained with Alcian blue for 1 h at RT. After removing the solution the cells were washed three times with water. Finally, samples were left to dry for up to 1 h at RT. Leaching was performed using 6M Guanidine-HCl (Sigma-Aldrich) for 1 h in the dark at RT. Measurements were made on a microplate absorbance reader (Mplex_Infinite M200; Tecan, Switzerland) at 650 nm.

### Statistical analysis

All the assays were performed on three biological replicates for each cell type. Statistical analysis was performed using Excel (Microsoft™) and SPSS (version 28.0.0.0.; IBM, UK). All data sets were tested to ensure Gaussian distribution using the Shapiro Wilks normality test and equal variance using Levene’s test for equality of variances. For comparisons of two groups Student’s t-tests were used. Data with more than two groups was analysed using one-way ANOVA with a Tukey’s *post-hoc* test. When data was not normally distributed, the data was log transformed or a non-parametric Kruskal Wallis test was performed followed by Dunn’s pairwise comparisons. In all cases a *p* value of < 0.05 was considered statistically significant.

## Results

### Inflammatory cytokines have negative effects on undifferentiated equine iPSCs

The iPSCs lines used in this study were characterized by their expression of pluripotency markers, capacity to generate embryonic bodies and undergo spontaneous differentiation into derivatives of endoderm, ectoderm and mesoderm when cultured in the absence of feeder cells, LIF and bFGF. (Supplementary Fig. 1). An overview of the experimental procedures is shown in Fig. 1.

**Figure 2.**
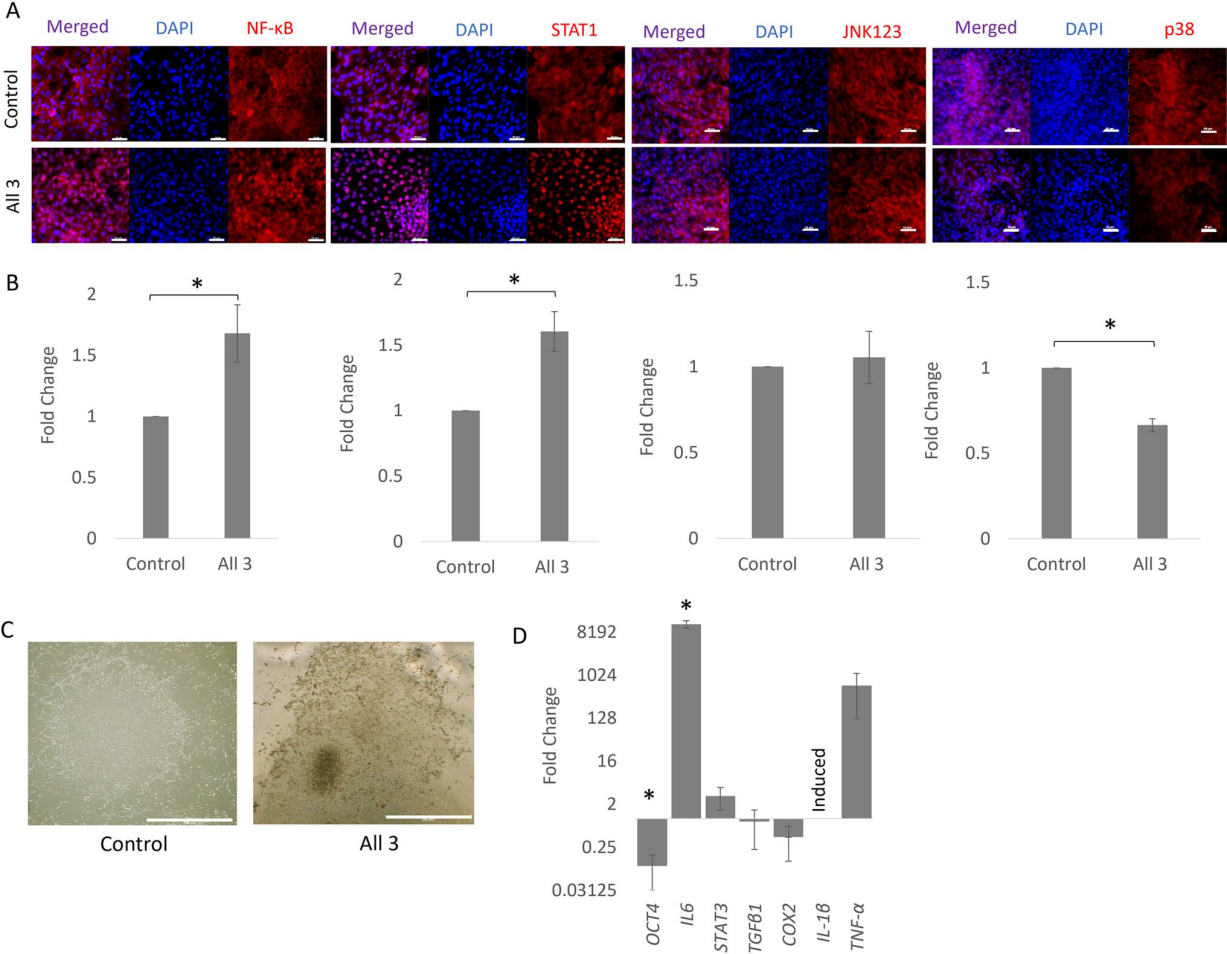
Response of undifferentiated equine iPSCs to inflammatory cytokine stimulation. (***A)*** Immunofluorescence staining of NF-κB, STAT1, JNK1/2/3 and p38 MAPK in equine iPSCs after 1 h of exposure to IL-1β, TNFα and IFN-γ (all 3) stimulation compared to the control (no stimulation). DAPI staining of the nucleus is shown in blue. Images are representative of three biological replicates. *Scale bar* = 50 µm. (***B)*** Quantification of the relative nuclear fluorescent intensity of NF-κB, STAT1, JNK1/2/3 and p38 MAPK after 1 h of cytokine stimulation (all 3) shown as a fold change compared to the control (no stimulation). *Error bars* represent the S.E.M from three biological replicates, *p < 0.05. (***C)*** Microscope imaging of iPSCs colonies after 72 h without (control) and with all three cytokines (all 3). Images are representative of three biological replicates. Scale bar = 200 µm. (***D)*** Fold change in gene expression in undifferentiated iPSCs cells following 72 h stimulation with all three cytokines compared to the control (no stimulation) and shown on a log2 scale. *Error bars* represent the S.E.M of three biological replicates, *p < 0.05.

Undifferentiated equine iPSCs show predominantly cytoplasmic staining of NF-κB P65 under non-stimulated conditions. In response to a combination of IL-1β, TNFα and IFN-γ, NF-κB P65 is translocated to the nucleus within 1 h and quantification of the nuclear staining is significantly increased. Similarly, in unstimulated equine iPSCs there is a low level of both nuclear and cytoplasmic STAT1. However, after stimulation with IL-1β, TNFα and IFN-γ there is significant accumulation of nuclear STAT1. In contrast, JNK1/2/3 shows a similar nuclear and cytoplasmic localisation in both stimulated and non-stimulated cells and p38 MAPK shows nuclear and cytoplasmic staining which decreases following stimulation with cytokines (Fig. 2*A* and *B*).

Although no overt change in cell morphology was observed following 1 h of cytokine stimulation, after 72 h of cytokine stimulation we observed considerable cell death within equine iPSC colonies, despite the feeder cell layer being unaffected (Fig. 2*C*). This coincided with a significant decrease in the pluripotency gene *OCT4* and a very large, significant increase in the inflammatory associated gene *IL-6* (over 8000-fold). There was also an induction of *IL-1β* and a large (over 300-fold), but not significant increase in *TNFα* (due to high levels of variability in the degree of change between the replicates). Other genes associated with inflammation such as *STAT3*, *TGF-β1* and *COX2* showed no, or small and not significant, changes in expression in response to 72 h of cytokine stimulation (Fig. 2D).

### Inflammatory cytokines induce translocation of NF-κB P65 and STAT1 and result in increased expression of inflammatory genes in spontaneously differentiated equine iPSCs

**Figure 3.**
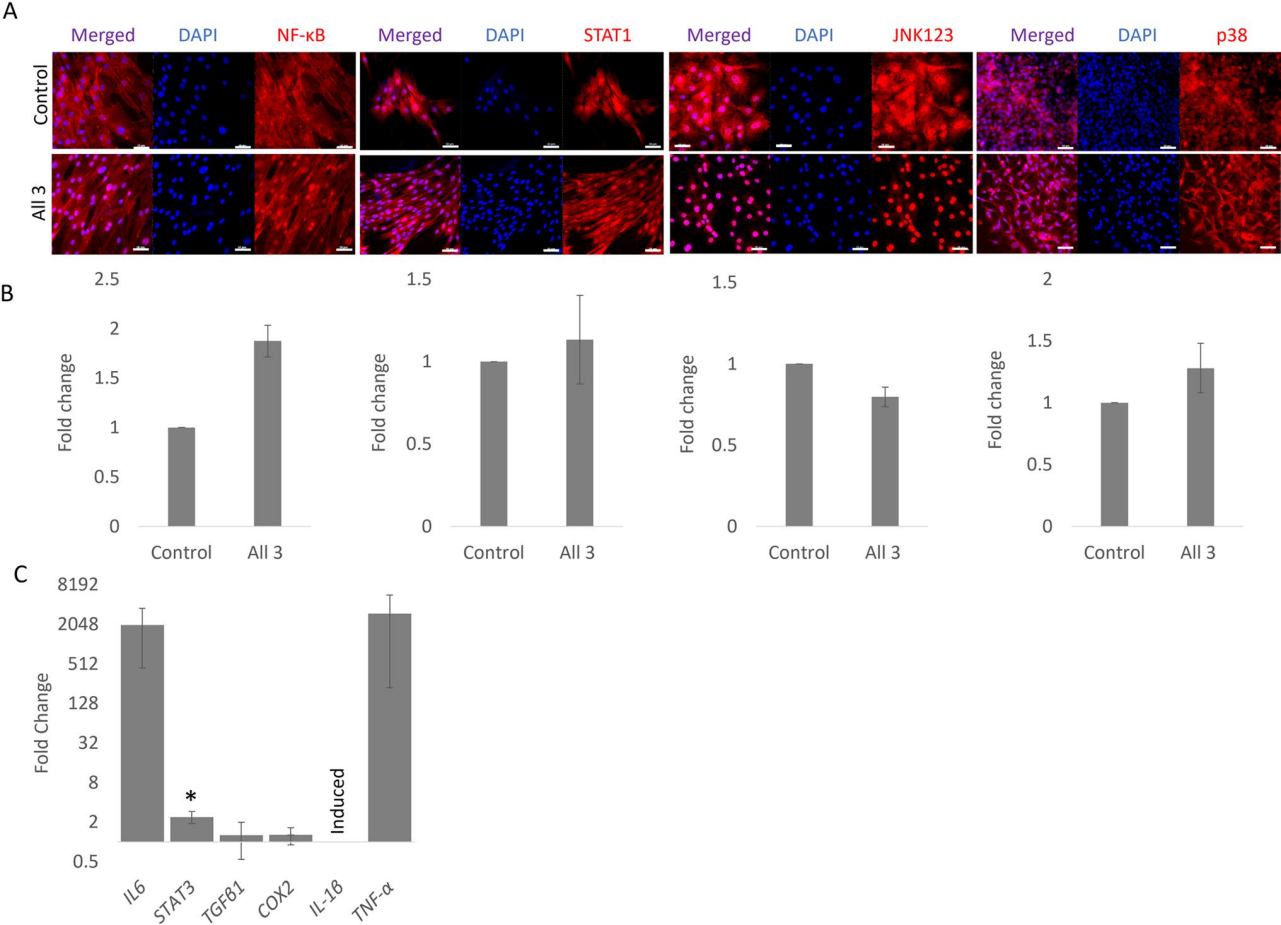
Response of spontaneously differentiated iPSCs to inflammatory cytokine stimulation. (***A)*** Immunofluorescence staining of NF-κB P65, STAT1, JNK1/2/3 and p38 MAPK in equine iPSCs that have been allowed to spontaneously differentiate for 10 days and then exposed to IL-1β, TNFα and IFN-γ (all 3) for 1 h compared to the control (no stimulation). DAPI staining of the nucleus is shown in blue. Images are representative of three biological replicates. *Scale bar* = 50 µm. (***B)*** Quantification of the relative nuclear fluorescent intensity of NF-κB P65, STAT1, JNK1/2/3 and p38 MAPK after 1 h of cytokine stimulation (all 3) compared to the control (no stimulation). *Error bars* represent the S.E.M from three biological replicates, *p < 0.05. (***C)*** Fold change in gene expression in spontaneously differentiated iPSCs cells following stimulation with all three cytokines compared to the control (no stimulation) and shown on a log2 scale. *Error bars* represent the S.E.M of three biological replicates, *p < 0.05.

We next allowed the iPSCs to spontaneously differentiate for 10 days into a mixed cell population by the removal of the feeder cells, LIF and bFGF. These conditions are sufficient to allow cells derived from all three germ layers to be produced ((Bavin *et al*. 2015) and Supplementary Fig. 1). Similar to the undifferentiated iPSCs, exposure to all three cytokines together produced an increase in nuclear NF-κB P65 (Fig. 3*A*). However, due to the variability between the replicates and across the coverslips (likely due to the heterogeneous differentiation) the quantification of nuclear staining showed no significant differences between conditions (Fig. 3*B*). STAT1 showed a predominantly nuclear localisation and was not affected by the cytokine exposure. JNK1/2/3 showed a strong nuclear and weaker cytoplasmic staining in the control conditions lacking cytokines, and a strong, more defined nuclear staining following exposure to all three cytokines. However, the intensity of nuclear staining was not quantitatively increased (Fig. 3*B*). p38 MAPK shows a fairly low level of nuclear and cytoplasmic staining under both stimulated and non-stimulated conditions (Fig. 3*A* and *B*).

Following 72 h of exposure to all three cytokines, we measured the expression of inflammation-associated genes. We saw very large increases in *IL6* and *TNFα* (over 2000-fold), but the variability between replicates meant that these changes were not statistically significant. The cytokine treatment also led to an induction of *IL-1β* and a small, but significant increase in *STAT3* expression. *TGF-β1* and *COX2* showed no changes in expression (Fig. 3*C*).

### Inflammatory cytokines activate STAT1 signalling in cartilage-like cells derived from equine iPSCs and lead to significant changes in gene expression

**Figure 4.**
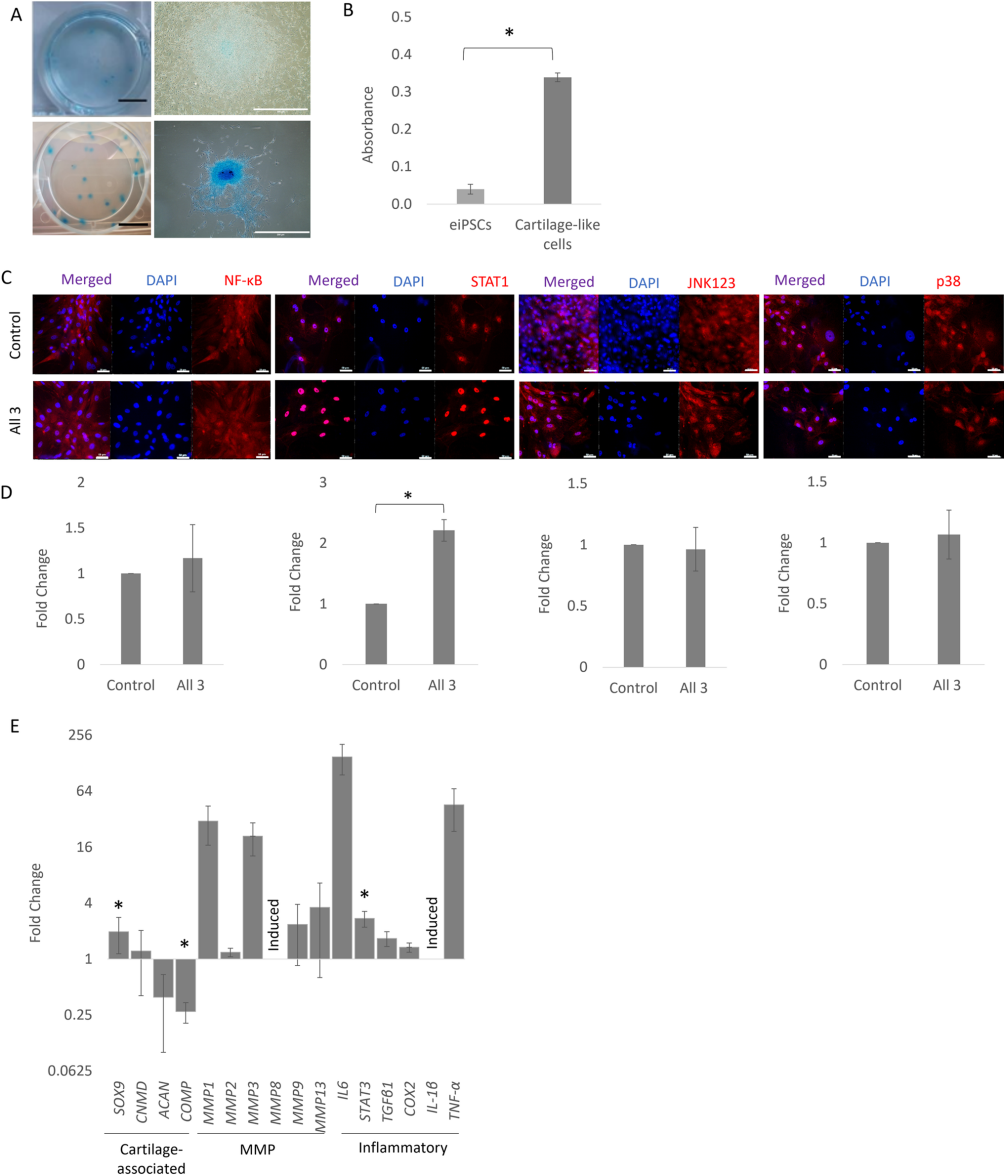
Response of cartilage-like cells derived from iPSCs to cytokine stimulation. (***A)*** Alcian blue staining of undifferentiated iPSCs (*top row*) and cartilage-like cells derived from iPSCs (*bottom row*). *Scale bars* = 1 mm (*left column*) and 200 µm (*right column*). (***B)*** Quantification of Alcian blue absorbance at 650 nm following stain leaching in undifferentiated iPSCs and iPSC-derived cartilage-like cells. Error bars represent the S.E.M from three biological replicates. *p < 0.05. (***C)*** Immunofluorescence staining of NF-κB P65, STAT1, JNK1/2/3 or p38 MAPK in cartilage-like cells derived from iPSCs after 1 h of cytokine stimulation (all 3) compared to the control (no stimulation). DAPI staining of the nucleus is shown in *blue*. Images are representative of three biological replicates. *Scale bar* = 50 µm. ***D ***Quantification of the relative nuclear fluorescent intensity of NF-κB P65, STAT1, JNK1/2/3 or p38 MAPK after 1 h of cytokine stimulation (all 3) compared to the control (no stimulation). *Error bars* represent the S.E.M of three measurements from each of three biological replicates. *p < 0.05. ***E*** Fold change in cartilage-associated, MMP and inflammatory gene expression in cartilage-like cells following stimulation with all 3 cytokines (IFN-γ, TNFα and IL-1β) compared to the no cytokine control and shown on a log2 scale. Induced = expression only detected following exposure to the cytokines and not in the control conditions. *Error bars* represent the S.E.M of three biological replicates, *p < 0.05.

Undifferentiated equine iPSCs show little Alcian blue staining. However, following 21 days of culture in the presence of TGF-β1 (and absence of feeder cells, LIF and bFGF) all iPSC colony outgrowths stained positively for Alcian blue indicating glycosaminoglycan deposition (Fig. 4*A*). Stain leaching and quantification demonstrated a significant increase in Alcian blue levels following differentiation (Fig. 4*B*). After 21 days of differentiation, the cartilage-like cells were exposed to the inflammatory cytokines. After 1 h of exposure, we observed a significant increase in nuclear STAT1, but no change in the nuclear protein levels of NF-κB P65, JNK1/2/3 or p38 MAPK (Fig. 4*C* and *D*).

After 72 h exposure to all three cytokines the expression of the cartilage associated gene *SOX9* showed a small, but significant increase. Whereas *ACAN* and *COMP* showed decreases in expression, which was significant for *COMP*. *CNMD* expression was unaffected. *MMP1* and *MMP3* showed robust, but not significant increases in gene expression, and *MMP8* was induced following cytokine exposure. In contrast, *MMP2*, *9* and *13* showed no or smaller changes in expression. *IL6* and *TNFα* showed large increases in expression but the differences were not significant and *IL-1β* expression was only detected following cytokine exposure. *STAT3* expression showed a small, but significant increase, and *TGF-β1* and *COX2* showed no changes in expression (Fig. 4*E*).

### Inflammatory cytokines induce translocation of NF-κB P65 and alter gene expression in iPSC-derived tendon-like cells

**Figure 5.**
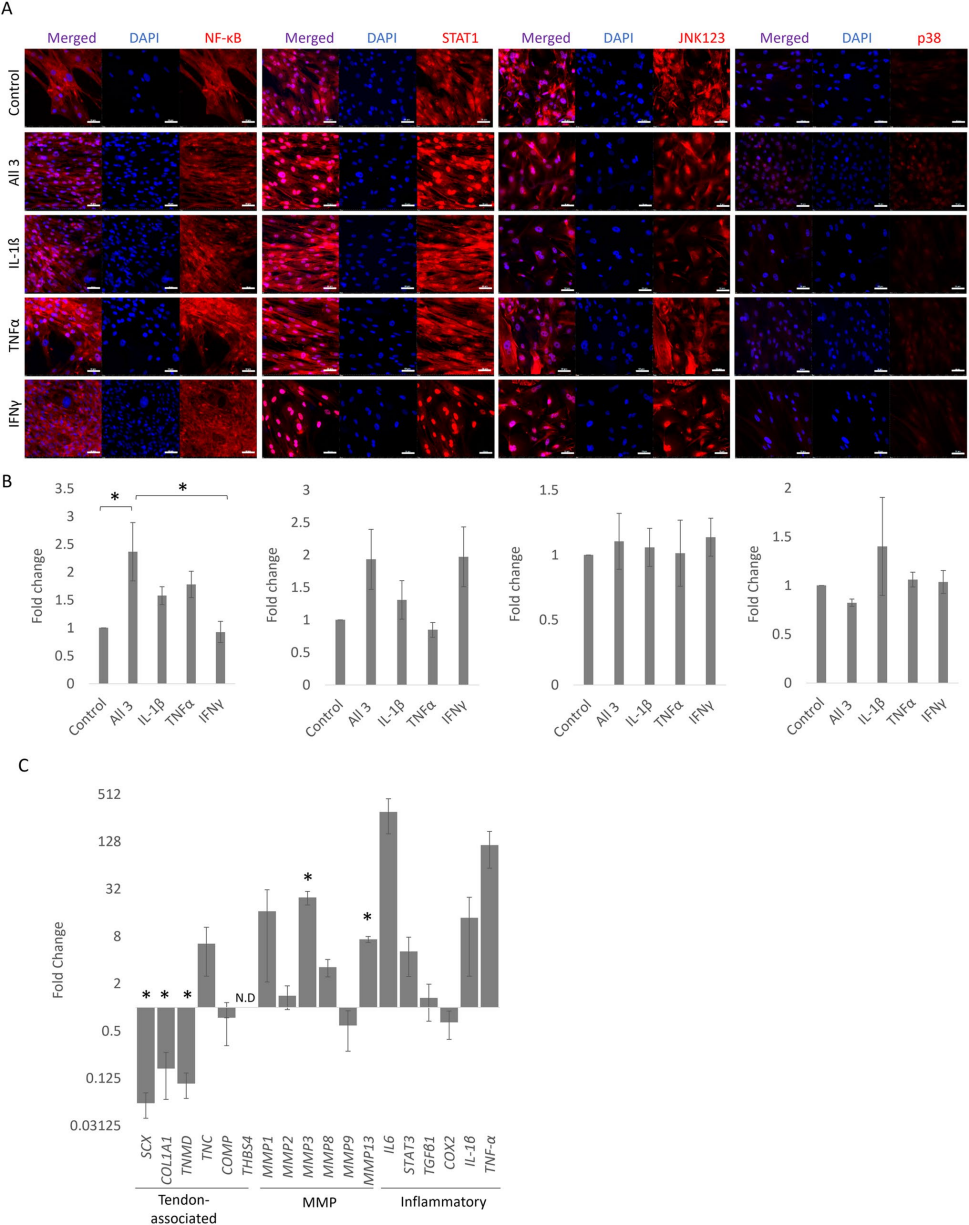
Response of tendon-like cells derived from equine iPSCs to cytokine stimulation. (***A)*** Immunofluorescence staining of NF-κB P65, STAT1, JNK1/2/3 or p38 MAPK in tendon-like cells derived from iPSCs after 1 h of cytokine stimulation (IL-1β, TNFα and/or IFN-γ stimulation) compared to the control (no stimulation). DAPI staining of the nucleus is shown in *blue*. Images are representative of three biological replicates. *Scale bar* = 50 µm. (***B)*** Quantification of the relative nuclear fluorescent intensity of NF-κB P65, STAT1, JNK1/2/3 or p38 MAPK after 1 h of cytokine stimulation (IL-1β, TNFα and/or IFN-γ) compared to the control (no stimulation). Error bars represent the S.E.M of three measurements from each of three biological replicates. *p < 0.05. (***C)*** Fold change in tendon-associated, MMP and inflammatory gene expression in tendon-like cells following stimulation with all 3 cytokines (IFN-γ, TNFα and IL-1β) compared to the no cytokine control for 72 h and shown on a log2 scale. N.D = not detected. *Error bars* represent the S.E.M of three biological replicates, *p < 0.05.

We have previously demonstrated that equine iPSCs upregulate tendon associated gene and protein expression following 14 days of culture in the presence of TGF-β3 (and absence of feeder cells, LIF and bFGF) (Bavin *et al*. 2015). Here, we demonstrate that this population of cells are capable of responding to inflammatory cytokines in a similar manner to that which we recently described for primary tenocytes isolated from the superficial digital flexor tendon of horses (Smith *et al*. 2023). In response to a 1 h exposure to all three cytokines, the iPSC-tendon-like cells exhibit a significant increase in NF-κB P65 nuclear staining (Fig. 5*A* and *B*). There is also a trend for a small increase in nuclear NF-κB p65 staining following IL-1β and TNFα exposure alone. In contrast, STAT1 undergoes a trend for increased nuclear staining following exposure to IFN-γ alone or all three cytokines together. JNK1/2/3 shows strong nuclear staining under control conditions which is not further altered by cytokine exposure, and p38 shows very weak staining under all conditions (Fig. 5*A* and *B*). This pattern is very similar to that which we observed in primary tendon cells (Smith *et al*. 2023).

72 h following exposure to all three cytokines, we observed a significant downregulation of tendon associated genes *SCX*, *COL1A1* and *TNMD* and a trend for an increase in *TNC*. *COMP* and *THBS4* expression levels were not changed (Fig. 5C). *MMP* expression was altered with a robust (but not significant) increase in *MMP1* and significant increases in *MMP3* and *MMP13*. Inflammatory gene expression was also altered with over a 200-fold increase in *IL6* and large increases in *IL-1β* and *TNFα*. But due to the variability in the scale of the fold change between biological replicates, these increases were not significant. Other genes associated with inflammation such as *STAT3*, *TGF-β1* and *COX2* showed no, or small and not significant changes in expression in response to 72 h of cytokine stimulation.

## Discussion

We have previously demonstrated that, like human and mouse ESCs and their derivatives (D’Angelo *et al*. 2017, 2018), equine ESC-derived tendon cells do not respond to the inflammatory cytokine IL-1β (McClellan *et al*. 2019a). Conflicting reports on the responsiveness of iPSCs and their differentiated progeny to inflammatory cytokines have been published (Lee *et al*. 2017; Hagman *et al*. 2019; Hyvärinen *et al*. 2019; Chen *et al*. 2020; Demine *et al*. 2020; Saraf *et al*. 2021) and to date, there are no reports examining this in equine iPSCs.

Here we demonstrate that undifferentiated equine iPSCs do respond to a combination of IL-1β, TNFα and IFN-γ. After 1 h they demonstrate increased nuclear staining of both NF-κB P65 and STAT1 and after 72 h they have a decreased expression of the pluripotency factor *OCT4* and increased expression of inflammatory associated genes including *IL-1β* and *TNFα*, which suggests a positive feedback loop. *IL6*, which is a known target gene of NF-κB (Liu *et al*. 2017), was also upregulated in response to inflammatory stimulation. *STAT3* expression is associated with inflammation and fibrosis, and is upregulated in response to IL6 signalling (Kasembeli *et al*. 2018). Nevertheless, we did not observe any changes in *STAT3* expression within the time frame studied. Future work should confirm if the increased *IL6* gene expression correlates with an increase in IL6 protein levels. Cytokine exposure also resulted in observable cell death after 72 h which did not occur in any of the differentiated cell types we studied. However, we did not perform any cell viability assays to quantify this. A previous report on undifferentiated human iPSCs found that these cells exhibited no response to TNFα alone or TNFα plus IFN-γ (Chen *et al*. 2020). We did not look at the effect of these specific conditions on the undifferentiated equine iPSC cells.

The spontaneous differentiation of the equine iPSCs resulted in a mixed population of cells, representing derivatives of all three germ layers. Similar results to the undifferentiated iPSCs were observed, with an increase in NF-κB P65 nuclear localisation, increased *IL6* and *TNFα* expression and induction of *IL-1β*. However, none of these increases were significant, likely due to the very heterogenous population of cells produced. In contrast, we did observe a small but significant increase in *STAT3*.

We then directed the iPSCs towards cartilage or tendon-like cells using TGF-β1/dexamethasone or TGF-β3 respectively. Following cartilage differentiation, we observed positive Alcian blue staining for glycosaminoglycans, and although we had expression of cartilage-associated genes, we did not quantify the heterogeneity of the resulting population. TGF-β1/dexamethasone has previously been shown to drive the chondrogenic differentiation of human iPSCs (Saitta *et al*. 2014), but a two stage protocol via a mesenchymal intermediate may increase the efficiency of differentiation (Diederichs *et al*. 2019) and this could be optimised in future work. Nevertheless, we were able to demonstrate that the iPSC-derived cartilage-like cells were able to respond to inflammatory cytokines with a significant increase in STAT1 nuclear localisation, and changes in cartilage-associated, *MMP* and inflammatory gene expression. We observed an increase in the early cartilage transcription factor *SOX9* and a decrease in the more mature cartilage markers *ACAN* and *COMP*. This may suggest that the efficiency of the differentiation is reduced in the presence of inflammatory cytokines, but we did not directly test this here. We also observed increases in *MMP, IL6*, *IL-1β*, *TNFα* and *STAT3* gene expression. Human iPSC-derived chondrocytes have also been shown to increase *IL6*, *MMP3* and *SOX9* expression in response to IL-1β (Lee *et al*. 2017). However, in this study, the authors demonstrated that the scale of the response was lower in iPSC-derived chondrocytes and juvenile chondrocytes than it was in adult chondrocytes. A limitation of our study is that we did not carry out the same experiments with primary equine chondrocytes to determine if the iPSC-cartilage like cells responded to inflammation to a similar degree to primary cells.

We have previously demonstrated equine iPSCs cultured for 14 days in the presence of TGF-β3 (and absence of feeder cells, LIF and bFGF) results in an increase in tendon-associated gene and protein expression (Bavin *et al*. 2015). Here, we have confirmed that tendon-associated genes are expressed following our differentiation protocol, but we did not measure protein expression. However, it is unlikely that we have a homogenous population of cells as we have previously shown they fail to contract a 3-D collagen gel; a measure of cell mediated matrix re-organisation (Bavin *et al*. 2015). The stepwise differentiation of human iPSCs to tendon cells has been reported previously (Nakajima and Ikeya 2021), but these approaches have not yet been applied to equine cells and accurate tendon cell markers are lacking (Li *et al*. 2021). Despite the probable heterogeneity within our population, here we show that equine iPSC tendon-like cells exhibit a similar response to that which we have recently observed in primary equine tendon cells (Smith *et al*. 2023). For example, both primary tenocytes and iPSC tendon-like cells have significantly increased nuclear NF-κB P65 staining after exposure to all three cytokines. In the primary tenocytes, this was also seen to occur in response to IL-1β or TNFα alone, but the increase in the iPSC tendon-like cells was not significant in these conditions (possibly due to the heterogenous nature of the differentiation). In primary tenocytes, nuclear STAT1 is significantly increased in response to IFN-γ exposure (Smith *et al*. 2023), and a similar trend is observed in the iPSC tendon-like cells, although the increase is not significant. In both the primary tenocytes and iPSC tendon-like cells no increase in JNK1/2/3 or p38 MAPK is observed in response to any of the inflammatory cytokines. However, a limitation of our study is that we only utilised immunofluorescence to determine nuclear localisation and in future studies other techniques, such as western blot, could be used to validate the results.

Similarly, in both the primary tenocytes and the iPSC tendon-like cells we observed significant decreases in tendon-associated genes such as *SCX* and *COL1A1* along with an increase in *TNC* and *MMP1, 3, 8* and *13*. However, the scale of the changes in the *MMP* genes is much larger in the primary tenocytes (over 100-fold for *MMP3* and *8* (Smith *et al*. 2023)). Again, this may reflect the heterogeneity of the iPSC tendon-like cell population. In this study we did not measure the expression of the tendon-associated proteins and further work is required to determine if the observed changes in gene expression are also reflected at the protein level. Here, we also examined the expression of inflammatory genes and found a similar pattern as for the undifferentiated and other differentiated iPSCs, with a large increase in *IL6*, *IL-1β* and *TNFa* and a smaller increase in *STAT3*. *COX2* is another target gene of NF-κB (Nakao *et al*. 2000) and has previously been shown to be increased in response to IL-1β and TNFα exposure in mouse, rat and human tenocytes (Oreff *et al*. 2021). Interestingly, this increase was not observed in either sheep or horse tenocytes (Oreff *et al*. 2021) and in our study we saw no increase in *COX2* expression in response to cytokine stimulation in any of the equine iPSC derived cell types investigated. TGFβ can drive fibrosis and is overexpressed during a tendon injury (Morita *et al*. 2016). IL-1β exposure of human tenocytes from diseased tissues leads to an upregulation in *TGF-β1* expression, but this does not occur in tenocytes isolated from healthy tissues (Morita *et al*. 2019). Similarly, we did not observe any changes in *TGF-β1* expression in our iPSC-derived tendon-like cells, or any of the other cell types studied.

Together, this work demonstrates that equine iPSCs both pre and post differentiation into multiple lineages are able to respond to the inflammatory cytokines IL-1β, TNFα and IFN-γ. This supports work using iPSCs from other species that have demonstrated cytokine responsiveness following lineage differentiation (Lee *et al*. 2017; Hagman *et al*. 2019; Hyvärinen *et al*. 2019; Demine *et al*. 2020; Saraf *et al*. 2021). However, it contrasts with our work on equine ESCs. While we have not yet examined the response of equine ESC-derived tendon cells to TNFα, we have previously shown that equine ESC-derived tendon cells do not respond to the inflammatory cytokine IL-1β (McClellan *et al*. 2019a). However, they are capable of responding to IFN-γ as an upregulation of MHC I was observed (McClellan *et al*. 2019b). We found that the lack of response to IL-β was due to a reduced IL1R1 expression, an increased expression of the decoy IL1R2 receptor and increased IL1 receptor antagonist protein gene expression (McClellan *et al*. 2019a). It is not clear if this reflects a lack of IL-1β receptor expression in the inner cell mass cells of horse blastocysts from which the ESCs are derived. However, in bovine embryos it has been shown that IL1R1 is present on trophectoderm cells, but not in inner cell mass cells (Correia-Álvarez *et al*. 2015). It would be of interest to compare cytokine receptor expression in horse ESCs and iPSCs before and after differentiation to understand the differences in the responsiveness of the cells to inflammatory stimulation.

The equine iPSCs used in this study have been characterised for their expression of pluripotency markers and ability to differentiate into derivatives of endoderm, mesoderm and ectoderm in vitro (Bavin *et al*. 2015; Baird *et al*. 2018, 2019). We have previously demonstrated that this method of reprogramming generates karyotypically normal iPSCs (Bavin *et al*. 2015), however the specific clonal iPSC lines used in this study did not undergo karyotyping. Furthermore, the ability of the iPSCs to form teratomas in vivo has not been tested and therefore it is possible that complete reprogramming to a pluripotent state equivalent to the ESCs has not been achieved. However, further work is also required to determine if ESC-derived cells develop responsiveness with longer differentiation protocols, or after in vivo transplantation is also required to determine what factors are necessary for the establishment of cytokine responsiveness during development.

Human adult tenocytes have been shown to establish an inflammation memory giving them an increased responsiveness to future stimuli (Dakin *et al*. 2017, 2018b). Our work on equine tenocytes (Smith *et al*. 2023), ESCs (McClellan *et al*. 2019a) and iPSCs in this study has investigated only the short-term effects of a single inflammatory cytokine stimulation. Future work to determine the longer term effects of both short-term, long term and repeated exposure to cytokines is required in order to better mimic the response of cells to acute and chronic inflammation.

## Conclusions

In conclusion, we have demonstrated that equine iPSCs and their differentiated progeny are negatively affected by short-term exposure to inflammatory cytokines which may have relevance for their clinical application.

### Supplementary Information

Below is the link to the electronic supplementary material.

Supplementary file1 (DOCX 1482 KB)

## Data Availability

All relevant data are within the manuscript and its supplementary information files.
