# Supplementary material for: Equine induced pluripotent stem cells are responsive to inflammatory cytokines before and after differentiation into musculoskeletal cell types
Source: In Vitro Cell Dev Biol Anim. 2023 Aug 15;59(7):514–27. doi: 10.1007/s11626-023-00800-3 (PMC10520172; doi:10.1007/s11626-023-00800-3)
Supplement: Supplementary file 1 — Supplementary file1 (DOCX 1482 KB) [file 11626_2023_800_MOESM1_ESM.docx]

**Supporting information**

**
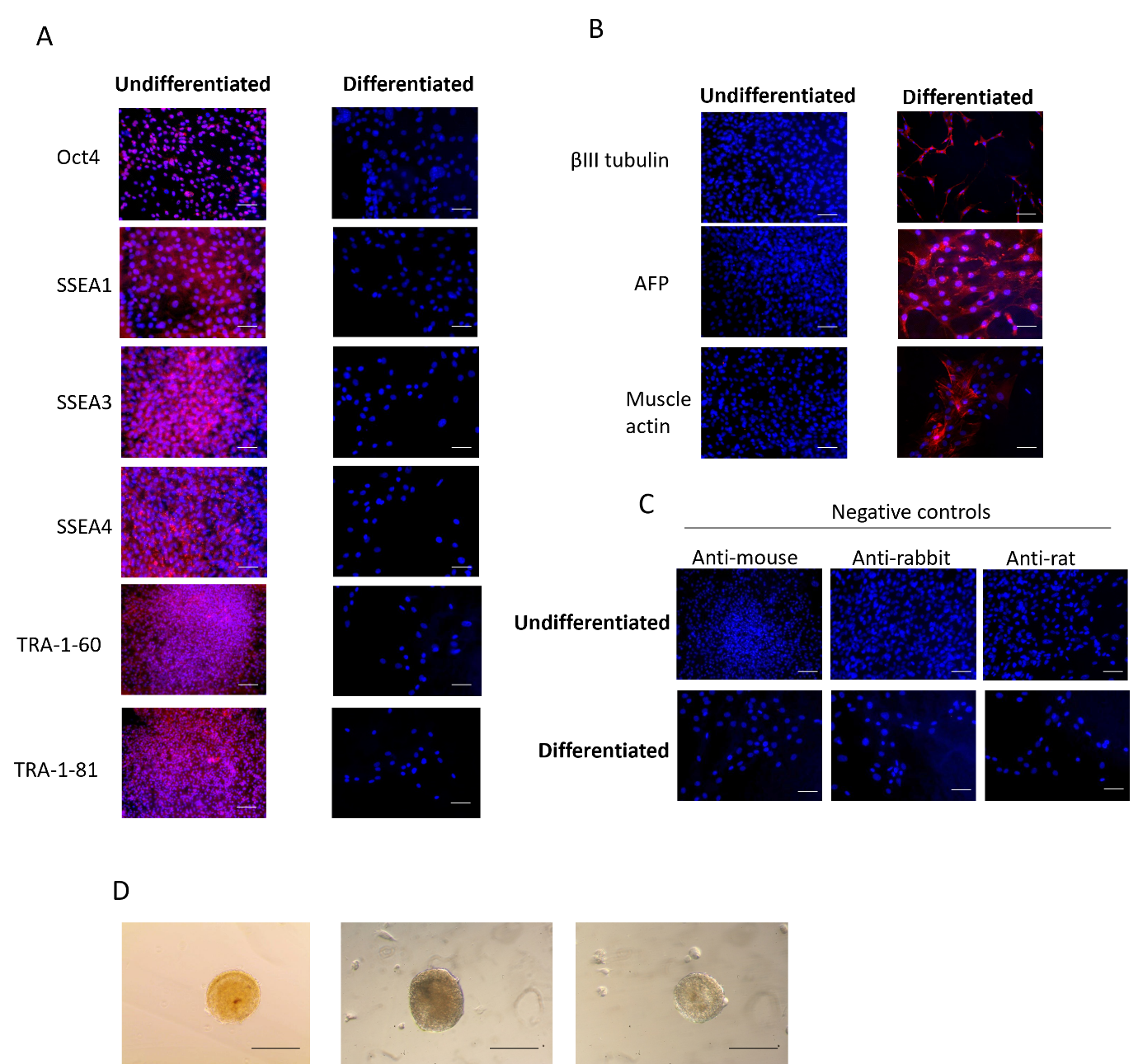
**

**Supplementary Figure 1.** Characterisation of the equine iPSC lines. (A) Undifferentiated equine iPSCs express pluripotency markers which are lost upon spontaneous differentiation. (B) iPSCs undergo spontaneous differentiation into the three germ layers after culture for 10 days in the absence of feeder cells, LIF and bFGF and express markers of differentiation which are not present in the undifferentiated cells. (C) Negative controls for the anti-mouse and anti-rabbit antibodies. DAPI staining of the nucleus is shown in blue. (D) Embryoid body formation by the equine iPSCs following culture on low attachment plates in the absence of feeder cells, LIF and bFGF. Scale bars A, B, C = 50 µm. Scale bars D = 400 µm.
